# Revisiting shyness and sociability: a preliminary investigation of hormone-brain-behavior relations

**DOI:** 10.3389/fpsyg.2014.01430

**Published:** 2014-12-23

**Authors:** Alva Tang, Elliott A. Beaton, Jay Schulkin, Geoffrey B. Hall, LouisA. Schmidt

**Affiliations:** ^1^Department of Psychology, Neuroscience and Behaviour, McMaster UniversityHamilton, ON, Canada; ^2^Department of Psychology, University of New OrleansNew Orleans, LA, USA; ^3^Department of Neuroscience, Center for the Brain Basis of Cognition and School of Medicine, Georgetown UniversityWashington, DC, USA; ^4^McMaster Integrative Neuroscience, Discovery and Study, McMaster UniversityHamilton, ON, Canada

**Keywords:** shyness, sociability, fMRI, temperament, cortisol, partial least squares (PLS)

## Abstract

Shyness and sociability are two fundamental personality dimensions that are conceptually and empirically orthogonal and are conserved across cultures, development, and phylogeny. However, we know relatively little regarding how shyness and sociability are represented and maintained in the brain. Here we examined neural responses to the processing of different types of social threat using event-related fMRI, the salivary cortisol awakening response (CAR), and sociability in young adults selected for high and low shyness. Shy adults who exhibited a relatively higher CAR displayed neural activity in putative brain regions involved in emotional conflict and awareness, and were more sociable. In contrast, shy adults who displayed a relatively lower CAR exhibited neural activity in putative brain regions linked to fear and withdrawal, and were unsociable. Results revealed no systematic brain responses to social threat processing that correlated with the CAR in non-shy adults. These preliminary results suggest that individual differences in waking morning cortisol levels may influence neural processes that facilitate either social approach or withdrawal among people who are shy. Findings are discussed in relation to their theoretical and clinical implications for moving beyond longstanding descriptive to explanatory models of shyness and sociability and for understanding individual differences in social behavior in general.

## INTRODUCTION

Over three decades ago, Cheek and Buss (1981) observed that some people are quiet and anxious in social situations for different reasons: some people are quiet and withdrawn because they are shy and have little need to affiliate with others (i.e., they are also unsociable), whereas others are inhibited and anxious because they are shy and desire to affiliate with others (i.e., they are also sociable). Cheek and Buss (1981) demonstrated that shyness and sociability were conceptually and empirically orthogonal and, contrary to popular belief, the two were not interchangeable. People who are shy are not necessarily unsociable. Cheek and Buss (1981) then developed short self-report measures of shyness and sociability and selected individuals who were high and low on shyness and sociability and had them interact during an unfamiliar social situation. The authors found that compared to their peers varying on high and low shyness and sociability, shy-sociable young adults exhibited significantly more anxious behavior, which were thought to originate from an approach–avoidance conflict, a combination of feelings of inhibition and desire to interact. Utility of the Cheek and Buss (1981) measurement model in predicting differential risk for psychopathology also has been demonstrated, with higher substance use and abuse among shy-sociable and higher depression and loneliness among shy-unsociable adolescents and young adults (for a review, see Schmidt and Buss, 2010).

To date, the independence of shyness and sociability has been replicated across developmental ages, including children (Asendorpf and Meier, 1993; Coplan et al., 2004; Coplan and Armer, 2007), adolescents (Page, 1990; Mounts et al., 2006), and adults (Eisenberg et al., 1995; Sheeks and Birchmeier, 2007; but see Bruch et al., 1989), across clinical populations (Goldberg and Schmidt, 2001; Jetha et al., 2009), and across cultures, including German (Czeschlik and Nurk, 1995), Portuguese (Neto, 1996), and Asian (Hussein et al., 2011) samples. The independence of these two basic dimensions is also ubiquitous across non-human animals (e.g., shyness/timidity and sociality/boldness; for a review, see Reale et al., 2007). Such universal behavioral manifestations of shyness and sociability across cultures, development, and phylogeny suggest the two personality traits are likely deeply rooted in our evolutionary history. Yet, relatively little is known beyond subjective and behavioral correlates of shyness and sociability, and still less about the representation of the two traits and mechanisms that maintain them in the brain.

### DISTINGUISHING SHYNESS AND SOCIABILITY ACROSS MULTIPLE PHYSIOLOGICAL LEVELS

In understanding the independence of shyness and sociability, separate studies have linked subtypes of shyness with specific biomarkers of stress vulnerability and reactivity. For example, studies using peripheral psychophysiological measures have demonstrated a unique autonomic pattern, with higher heart rate and lower vagal tone (correlates of stress reactivity) in shy-sociable children in their everyday environments (Asendorpf and Meier, 1993) and in shy-sociable young adults during the anticipation of unfamiliar social interactions, relative to their shy-unsociable counterparts (Schmidt and Fox, 1994). Studies using electrocortical measures have identified a distinct pattern of greater relative right frontal EEG asymmetry at rest (a brain correlate of stress vulnerability and avoidant behavior) across both shy-sociable and shy-unsociable young adults. What distinguished the two groups was the pattern of absolute activity in the left prefrontal cortex (PFC; Schmidt, 1999): greater left PFC activity (a brain correlate of approach behavior) was observed in the shy-sociable compared with the shy-unsociable group. Replication of similar frontal EEG asymmetry patterns at rest in clinical samples of outpatients diagnosed with schizophrenia across cultures who were shy and social (Jetha et al., 2009; Hussein et al., 2011) indicate the possibility of a conserved neural mechanism underlying these brain-behavior relations, irrespective of disease state and cultural influences.

These different patterns of brain and peripheral activity in shy-sociable and shy-unsociable individuals are presumably influenced by differential activity of corticolimbic neural networks that may precipitate different emotion and stress regulation strategies during social interactions. This notion is suggested by differential activity of prominent regions in corticolimbic networks, including the medial PFC, amygdala, hippocampus, and hypothalamic nuclei, which overlap in the modulation of emotion and stress regulation, and threat processing (Dedovic et al., 2009). For example, fMRI studies have found that during social threat processing, shy adults elicited hyperactivity of the amygdala to unfamiliar faces (Schwartz et al., 2003; Beaton et al., 2008), and differential amygdalar (Hardee et al., 2013) and medial PFC functional connectivity (Tang et al., under review) to angry faces among shy individuals. As well, changes in the hypothalamic-pituitary-adrenal axis (HPA-axis) and hormones (e.g., cortisol) involved in stress vulnerability and reactivity may arise in shy individuals because cortisol regulates its own release by binding to its receptors that are densely distributed in the corticolimbic system (Feldman et al., 1995; Herman et al., 2005). One measure that captures these dynamic cortisol changes is the cortisol awakening response (CAR).

The CAR, characterized as the peak in cortisol secretion within the first hour after awakening, is a specific component of the cortisol diurnal rhythm that is present in 75% of the population (Wust et al., 2000) across children, adolescents, adults, and older adults (Pruessner et al., 1997). The CAR appears to be the most reliable in predicting trait-related individual differences in personality and stress regulation, rather than state-related differences (Hellhammer et al., 2007; Fries et al., 2009). In an examination of the diurnal cortisol pattern across the day, the CAR has been demonstrated to capture the greatest and most reliable differences in relation to shyness (Beaton et al., 2013). Moreover, increases in morning cortisol are presumed to be adaptive in most individuals as one function is to prepare the individual psychologically and physically for the upcoming day, while decreases in CAR might reflect and signal maladaptive behavior in some individuals (Fries et al., 2009).

Interestingly, both high and low cortisol levels measured upon waking (i.e., the CAR), at baseline, and in response to stress have been found among individuals who are shy (for a review, see Beaton et al., 2013). High basal morning salivary cortisol has been reported in temperamentally shy preschoolers (Kagan et al., 1988; Schmidt et al., 1997) and increased salivary cortisol reactivity to social stress has been observed in temperamentally shy early school age children. However, high and low baseline salivary cortisol levels have been reported in temperamentally shy individuals in middle childhood (Schmidt et al., 1999, 2007) and adulthood (Bell et al., 1993; Beaton et al., 2006, 2013).

High and low cortisol levels suggest complex functional implications for regulating social behavior. For example, high and increases in basal cortisol levels have been observed in shy and inhibited children (Kagan et al., 1988) and older adults (Bell et al., 1993) as well as socially dominant, competent, bold, and exuberant children (Gunnar, 1994) and dominant non-human primates (e.g., Muller and Wrangham, 2004). In contrast, low and decreases in cortisol have been found in socially competent children as well as shy adults (Beaton et al., 2013) and some profiles exposed to chronic stress and characterized as depressed and socially withdrawn (Gunnar and Vazquez, 2001).

Given the release of cortisol mobilizes energy for facing physical and social challenges, increases in cortisol levels may serve as a response to a stressor or the facilitation of approach behavior as observed in people who are social and outgoing. On the other hand, low and decreases in cortisol levels may reflect effective emotion regulation or the facilitation of withdrawal behavior and adaptation of the adrenocortical system to a life course of dealing with chronic fear and stress (e.g., Beaton et al., 2013). Accordingly, it is possible that individual differences in the CAR may be associated with brain processes that facilitate either social approach or withdrawal among people who are shy.

### THE PRESENT STUDY

Here we conducted a preliminary investigation to examine whether the relation between individual differences in the salivary CAR and neural responses to different types of social threat processing was associated with individual differences in shyness and sociability. While in a MR scanner, adults selected for high and low shyness viewed and discriminated pairs of congruent (angry/angry) and incongruent (angry/neutral) faces, representing imminent and ambiguous social threat, respectively, as same or different. The salivary CAR was measured across three consecutive mornings. First, to address whether individuals with high and low shyness varying in the CAR process social threat differently, partial least squares (PLS) was performed to identify brain-cortisol correlations simultaneously. Because a latent pattern of brain activity emerged as a function of high versus low CAR within the shy group that suggested relative differences in the engagement of two sets of brain regions to both types of social threats, assessment of how shy individuals varying in sociability were distributed across the two relatively different sets of regions was followed.

We predicted that shy individuals who exhibited a relatively higher CAR would display activation in putative brain regions with modulatory roles in conflict monitoring and approach-withdrawal conflict [e.g., the anterior cingulate cortex (ACC)] during threat processing and would be more sociable. In contrast, shy individuals who exhibited a relatively lower CAR would display activation in putative brain regions involved in fear and withdrawal (e.g., the amygdala) and would be less sociable.

## MATERIALS AND METHODS

### PARTICIPANT SELECTION

Twenty four right-handed participants were selected from 152 (61 males, *M* age = 19.74 years; and 91 females, *M* age = 20.41 years) undergraduate students for high (*n* = 12; five female and seven male; upper 25%) and low (*n* = 12; four female and eight male; bottom 25%) shyness based on their responses on the Cheek and Buss (1981) Shyness Scale. The groups did not significantly differ in age, *t*(22) = 0.67, or gender ratio, *χ*^2^(1) = 1.50, *ns*. Participants were screened for history and/or current mental illness, learning disability, and use of medication that acts on the central nervous or adrenocortical systems 2 weeks prior to the experiment.

All participants completed a series of questionnaires [e.g., Cheek and Buss, 1981 Shyness (e.g., “I am inhibited in social situations”) and Sociability (e.g., “I am socially outgoing”) Scales] as part of a larger study investigating the neural (Beaton et al., 2008) and endocrine (Beaton et al., 2013) correlates of social anxiety. Course credit was given to each participant for their voluntary participation in the initial screening and an additional $100 to the 24 participants, who were selected and participated in the larger study. The experiment was conducted with approval from the McMaster University Health Sciences and St. Joseph’s Healthcare Research Ethics Board.

### SALIVARY CORTISOL

#### Collection and procedures

To obtain reliable estimations of cortisol measures reflective of stable individual differences, participants collected morning salivary cortisol measures across three separate non-stressful days. Saliva collection kits were given to participants. These kits included: (a) 15 sterile 1.5 ml Nalgene cryotubes that were color coded according to the requested saliva sampling time, (b) a form to record perceived stressful life events (i.e., event description and time of day) during the collection period, and (c) a Palm III personal data assistant (PDA; Palm Inc., Sunnyvale, CA, USA). Participants approximated and inputted their typical wake/sleep schedule into the PDA to set alarms that prompted saliva collection. Participants were prompted to expectorate at least 0.75 ml of saliva at five time points, each distinguished by a different colored cryotube, across 3 days: (a) upon awakening but before arising from bed, corresponding to a baseline; (b) 60 min post-awakening; (c) 8 h post-awakening, corresponding to the afternoon; (d) 10 h post-awakening, corresponding to the early evening; and (e) bedtime. These samples were stored in participants’ home refrigerators while under their control. Subsequent to returning saliva collection to the laboratory, samples were stored at -80°C until assayed.

There was a high degree of inter-individual variability (Wust et al., 2000) in the start-of-day and length-of-day measurement period, which is typical of young adult university attendees. Twenty participants consecutively collected samples from Tuesday to Thursday, while two participants collected samples on Tuesday, Wednesday, and Friday. To maintain ecological validity, collection of multiple salivary samples across multiple days was designed to achieve reliable estimates of cortisol production and CAR that are reflective of the two traits, shyness and sociability, rather than state factors (Hellhammer et al., 2007). All participants reported being free of medication and were asked to refrain from consumption of alcohol, and to refrain from smoking or consuming any caffeine, dairy, or citrus juices 2 h before collecting saliva samples. Finally, participants were urged to adhere to their typical sleep schedules.

#### Enzyme-linked immunoassay (EIA)

Hormone assays from saliva were conducted at the Neuroendocrinology Laboratory at St. Joseph’s Healthcare in Hamilton, ON, Canada. Samples were thawed, mixed, and centrifuged for 15 min at 1500 *g*. A commercial competitive enzyme immunoassay kit optimized for saliva (HS-Cortisol High Sensitivity, Salimetrics®, LLC, State College, PA, USA) was used to derive salivary cortisol concentrations. Standards, controls, and samples were assayed in triplicate at a volume of 25 μl. All samples with a coefficient of variability that exceeded 15% were repeated (*n* = 9) as a singleton on another plate. The mean of the triplicates was then used in subsequent analyses. Each plate included manufacturer-supplied salivary cortisol controls and the coated plate was incubated at room temperature for 1 h in the presence of 200 μl of enzyme conjugate. Plates were then washed four times by hand. The plates were read within 10 min of adding the stop solution at 492 and 450 nm, the optical density difference between the two readings was measured using a Multiskan Ascent Microplate reader, (Thermo Electron Corporation, Milford, MA, USA). Cortisol concentrations were determined by interpolation using a 4-parameter sigmoid minus curve it software program (Multiskan Ascent): inter-assay variation = 5.08%; intra assay = 3.50%; and sensitivity = 0.08 nmol/l.

Of particular interest to the present study was the peak response of morning salivary cortisol, the CAR, which was derived from the difference between the awakening and 60 min post-awakening saliva samples across three mornings. The mean for each participant was used in further analyses.

### SOCIAL THREAT PROCESSING TASK

Visual stimuli consisted of photographs of angry and neutral faces obtained from Ekman and Friesen’s (1976) affective faces dataset. Photographs were modified to standardize all parameters including grayscale conversion, size, contrast, and luminosity adjustments. Participants completed the task within the MR scanner: in an event-related design, participants viewed pairs of faces which were congruent, angry/angry (*n* = 12 trials), or incongruent, angry/neutral (*n* = 13), and discriminated the two facial expressions of the same person as same or different by pressing buttons on a response box. The congruency design was to capture imminent (congruent) or ambiguous (incongruent) aspects of social threat, because they are particularly salient to individuals who are shy (Tang et al., under review) and probe different types of threat processing as they are believed to be supported by different neural networks (LeDoux, 2013). In each trial, a pair of faces was presented side-by-side for 2700 ms, followed by a jittered fixation cross varying in duration (2700–10800 ms).

### fMRI DATA ACQUISITION AND PREPROCESSING

Anatomical and functional images were collected using a General Electric 3-Tesla, whole-body short bore scanner with eight parallel receiver channels (General Electric, Milwaukee, WI, USA). T1-weighted anatomical images were acquired with a 3D volume spoiled gradient recalled (SPGR) pulse (124 axial slices, 1.5 mm thick, FOV = 240 mm, TR/TE = 86/21 ms, 12° flip angle, 512 × 512 matrix). Functional images were obtained using a T2*-weighted gradient-echo EPI sequence (28–38 axial slices, 4 mm thick, FOV = 240 mm, TR/TE = 2700/35 ms, 90° flip angle, 64 × 64 matrix), starting at the cerebral vertex and included the entire cerebrum and greater parts of the cerebellum.

Preprocessing of the dataset was performed in the Analysis of Functional Neuroimages (AFNI; Cox, 1996). The first two volumes of functional images were discarded to allow for stabilization of the magnetic field gradients. Preprocessing procedures included: reconstruction, slice timing correction for the time discrepancy between the first and final acquired slices, and rigid motion correction using 3D Fourier transform interpolation. To allow group comparisons, images were spatially normalized to the Montreal Neurological Institute (MNI; MNIavg152) spiral template, and smoothed using an 8 mm full width-half maximum isotropic Gaussian filter. The transformed images resulted in 4 mm × 4 mm × 4 mm isotropic voxels.

### DATA ANALYSIS

#### Partial least squares (PLS) analysis (cortisol and brain)

To address which corticolimbic regions correlated most strongly with morning salivary cortisol change scores, we examined between group (shy versus non-shy) differences in brain-cortisol correlations for two types of threat (imminent versus ambiguous) processing by conducting a behavioral-PLS (although we used a cortisol-PLS analysis, the word “behavior” is retained here as this is the convention in the literature; for a full description on the method; see McIntosh et al., 2004). This data-driven multivariate technique assumes that brain function results from coordinated activity between distributed regions and has been found to be sensitive to detecting task-related whole-brain patterns of blood-oxygen-level-dependent (BOLD) activity and brain-behavior relations (e.g., McIntosh et al., 2004; Turner et al., 2011). Primary advantages of PLS include the abilities to handle a large number of variables and high collinearity among variables in fMRI datasets and to derive whole-brain patterns of activity or brain maps that are predictive of cortisol patterns, which cannot be achieved by examining correlations between univariate voxel-wise changes in neural response of a predefined small number of regions and cortisol. Overall, PLS operates on the normalized and mean-centered covariance matrix between BOLD activity and cortisol data to extract Latent Variables (LVs) that capture across participants and within-condition brain-cortisol correlations for each of the two types of threat conditions (imminent and ambiguous) and two groups (shy and non-shy).

To begin, corresponding brain activity, and cortisol data are organized in two matrices. To put the matrix configuration of brain activity in view: experimental conditions are stacked and each row represents the data of each participant within each condition. There are *n* × *k* rows, with *n* participants and *k* conditions. Columns contain the hemodynamic response function (HRF) signal intensity for each voxel at each time point or lag in the temporal window after stimulus onset for each trial (i.e., given the TR = 2.7 s, there are six temporal lags to account for an HRF of ∼18 s). Accordingly, the HRF for each trial is broken into six columns, each representing the intensity difference from trial onset for each voxel. For example, the first column is the intensity for voxel one at lag one, the second column is the intensity for voxel one at lag two, and so on. There are *m* × *t* columns, with *m* voxels and *t* time points. To increase signal to noise ratio, normalization helps maintain the amplitude of BOLD signals (i.e., baseline correction) and allows trials within a condition to be averaged. These values are then expressed as voxel-by-voxel deviations from an across participants within-condition mean-centering procedure to create a normalized mean-centered covariance matrix.

Optimal least squares of fit to the crossblock part of the covariance between BOLD signal and cortisol are simultaneously computed across the entire data structure in time and space. Singular value decomposition (SVD) is then applied on the brain-cortisol covariance matrix to derive a set of orthogonal singular vectors or LVs and to produce three new matrices: (a) singular values, (b) task saliences, and (c) voxel saliences. The identified set of LVs are similar to eigenvectors in principal component analysis (PCA), in that both solutions capture the maximal covariance in two datasets and account for the original matrix in a decreasing order of magnitude. Moreover, because PLS solutions are additionally constrained to the part of the covariance structure that is attributed to the exogenous measures, the identified LVs here explain brain activity that is relevant to cortisol, threat conditions, and groups.

Singular values for each LV are used to calculate the proportion of the matrix accountable by each LV (i.e., the crossblock covariance). Each LV consists of a pair of vectors relating brain activity and the identified design components: task saliences are the degree to which a condition or group is related to the pattern demonstrating the relations between cortisol and/or experimental effects that are most related to differing signals in the identified set of voxels. Voxel saliences are weights at each voxel that produce patterns of a set of voxels that as a group covary with cortisol across groups and/or conditions, in the least squared sense.

The dot product of individual participant’s image volume and the saliences on a particular LV results in individual brain scores, which indicate the absolute degree to which a participant expresses the patterns in a particular LV. The brain score is conceptually similar to a factor score from factor analysis. Correlation between cortisol and brain scores across participants with each scan produces scan profiles, which are proportional to the singular profiles from SVD but permit simpler interpretations because they are correlations. These scan profiles are represented by scatter plots. Should the scan profiles indicate a similar correlation across tasks or groups, then salient voxels in the singular image would display a similar correlation with cortisol across tasks or groups. However, should the scan profiles differ between tasks or groups, then the singular image would reflect a task or group difference in brain-cortisol correlations.

Finally, cross-validation methods including 700 permutation tests and 350 bootstrap resampling estimations allowed statistical inference of LVs and generalization to new observations (McIntosh et al., 2004). Permutation tests determine the significance of each LV by ensuring the effects in a given LV significantly depart from random data: random orders of the conditions are assigned to each participant, PLS is recalculated for each new re-ordering and the probability of the permuted singular values exceeding the observed singular values is calculated. The probability for each LV is an index of whether it is retained. With the retained significant LVs, bootstrap estimations of standard errors (SEs) are used to assess the stability of voxel saliences that show reliable condition effects and/or cortisol relations: each participant is assigned the same order of experimental conditions. Because voxel saliences are calculated on the entire brain in a single step, correction for multiple comparisons is not necessary. Reliability assessment of individual voxels is specified by the ratio of the salience value to the bootstrap SE [bootstrap ratio (BSR) presented in **Table 1**], which are approximately equivalent to *z*-scores. Voxel saliences presented in **Table 1** and **Figure 2** were considered reliable, because the ratio of the observed to the estimated effects on bootstrap testing was greater than 3, corresponding to an approximate probability of *p* < 0.003.

**Table 1:** Reliability map of activation: stable voxels for the Latent Variable (LV) from the PLS analysis with cortisol as a correlate for the two groups (shy, non-shy) and two threat conditions (imminent, ambiguous).

		Talairach coordinates						
BSR	Laterality	X	Y	Z	Brain region	BA	Cluster size	Lag
**Positive saliences**
4.04	L	-5	6	58	Superior Frontal Gyrus	6	24	3
3.95	L	-9	49	36	Superior Frontal Gyrus	8	24	5
6.61	L	-31	11	-10	Inferior Frontal Gyrus*	13	114	4
5.14	L	-38	26	-9	Inferior Frontal Gyrus	47	14	3
3.72	R	37	30	-8	Inferior Frontal Gyrus	47	21	4
4.96	L	-5	41	36	Medial Frontal Gyrus*	6	137	4
5.16	L	-1	-2	61	Medial Frontal Gyrus*	6	60	5
4.52	R	2	-18	71	Medial Frontal Gyrus	6	16	5
5.06	L	-39	-5	46	Middle Frontal Gyrus*	6	131	4
6.81	L	-50	7	43	Middle Frontal Gyrus	6	18	4
4.42	R	32	11	38	Middle Frontal Gyrus	8	11	5
7.69	R	6	-24	49	Paracentral Lobule*	6	73	3
7.88	R	10	-24	49	Paracentral Lobule*	6	267	4
4.35	L	-46	-9	6	Precentral Gyrus	6	18	3
3.79	L	-28	-20	45	Precentral Gyrus	4	10	3
3.53	L	-35	-17	60	Precentral Gyrus	4	20	4
4.20	R	47	-40	16	Insula	13	17	5
3.54	L	-31	-8	-12	Amygdala		12	4
5.75	L	-31	-26	-20	Parahippocampal Gyrus	35	42	5
4.39	R	29	-26	-16	Parahippocampal Gyrus	36	22	4
8.05	R	17	-55	10	Posterior Cingulate*	30	353	4
3.87	R	10	-52	25	Posterior Cingulate	31	10	3
6.43	R	32	-23	35	Postcentral Gyrus*	2	314	5
5.91	L	-39	-26	34	Postcentral Gyrus	2	31	5
4.23	R	32	-43	51	Inferior Parietal Lobule*	40	51	3
3.88	L	-31	-38	40	Inferior Parietal Lobule	40	18	2
3.45	L	-39	-53	42	Inferior Parietal Lobule	40	11	4
4.14	L	-9	-70	52	Superior Parietal Lobule	7	14	5
3.92	L	-24	-46	47	Precuneus	7	10	4
4.54	R	17	-77	45	Precuneus	7	11	5
3.99	R	21	-72	31	Precuneus	31	10	4
3.91	R	25	-75	23	Precuneus	31	21	5
6.95	L	-57	-5	3	Superior Temporal Gyrus*	22	419	4
4.22	L	-57	-1	3	Superior Temporal Gyrus	22	11	5
9.06	R	36	-29	13	Transverse Temporal Gyrus*	41	864	4
6.90	R	36	-25	13	Transverse Temporal Gyrus	41	34	3
5.58	R	36	-49	-14	Fusiform Gyrus*	37	55	5
4.71	R	47	-70	6	Middle Occipital Gyrus*	37	65	4
4.49	L	-28	-78	12	Middle Occipital Gyrus	19	20	5
4.32	L	-24	-78	19	Cuneus	18	12	3
5.05	L	-20	-78	19	Cuneus	18	34	4
							
3.57	R	6	-74	9	Cuneus	23	10	3
6.14	R	6	-47	4	Culmen*		587	5
6.61	L	-12	-37	-17	Culmen		21	4
4.23	L	-27	-64	-20	Declive		24	5
**Negative saliences**
-4.70	L	-5	-40	62	Paracentral Lobule	5	12	1
-5.04	L	-20	41	0	Anterior Cingulate	32	24	2
-4.20	L	-16	37	-1	Anterior Cingulate	32	12	3
-4.85	R	10	33	-1	Anterior Cingulate	24	24	1
-7.18	R	7	33	3	Anterior Cingulate	24	25	2
-5.58	L	-16	25	9	Caudate Body		11	5

This map of activation in relation to cortisol patterns consists of brain regions with the most reliable saliences. BSR, bootstrap ratio, the salience value over the bootstrap SE, >±3.0 (corresponding approximately to *p*< 0.003), with a minimum cluster of 10 voxels. Laterality: L, Left; R, Right. Peak voxels are reported in Talairach coordinates, X, Y, Z. Brain regions* indicate cluster size ≥50. BA, Brodmann Area. Lag, temporal lag (accounting for the slow blood-oxygen-level-dependent response) window in which the voxel was found. Regions identified with positive saliences are reliably related to shy adults with a lower morning cortisol change; regions identified with negative saliences are reliably related to shy adults with a higher morning cortisol change in response to both Angry/Angry and Angry/Neutral conditions (see **Figure 2**).

All brain regions presented in the table exceed a minimum threshold of cluster size ≥10 voxels. Peak voxel locations are reported in corresponding Talairach coordinates. Conversion from MNI coordinates to match the Talairach atlas was performed with the icbm2tal transform in GingerALE (Lancaster et al., 2007). Due to technical and equipment problems, functional images of two non-shy participants were unusable, consequently, all results are based on 10 non-shy and 12 shy participants.

#### Chi-square analysis (cortisol and sociability)

To test the hypothesis of whether there were systematic cortisol profiles related to different levels of sociability within shyness, a median split was applied to the Cheek and Buss (1981) sociability scores and CAR change scores to create two levels in each variable: (a) high versus low sociability and (b) high versus low waking morning salivary cortisol levels within the shy group. A chi-square test of independence was then performed to examine the relation between CAR change scores (high versus low) and sociability levels (high versus low) among shy individuals.

## RESULTS

### RELATIONS BETWEEN MORNING SALIVARY CORTISOL CHANGE AND BRAIN SCORES (PLS) AMONG SHY ADULTS

**FIGURE 1:**
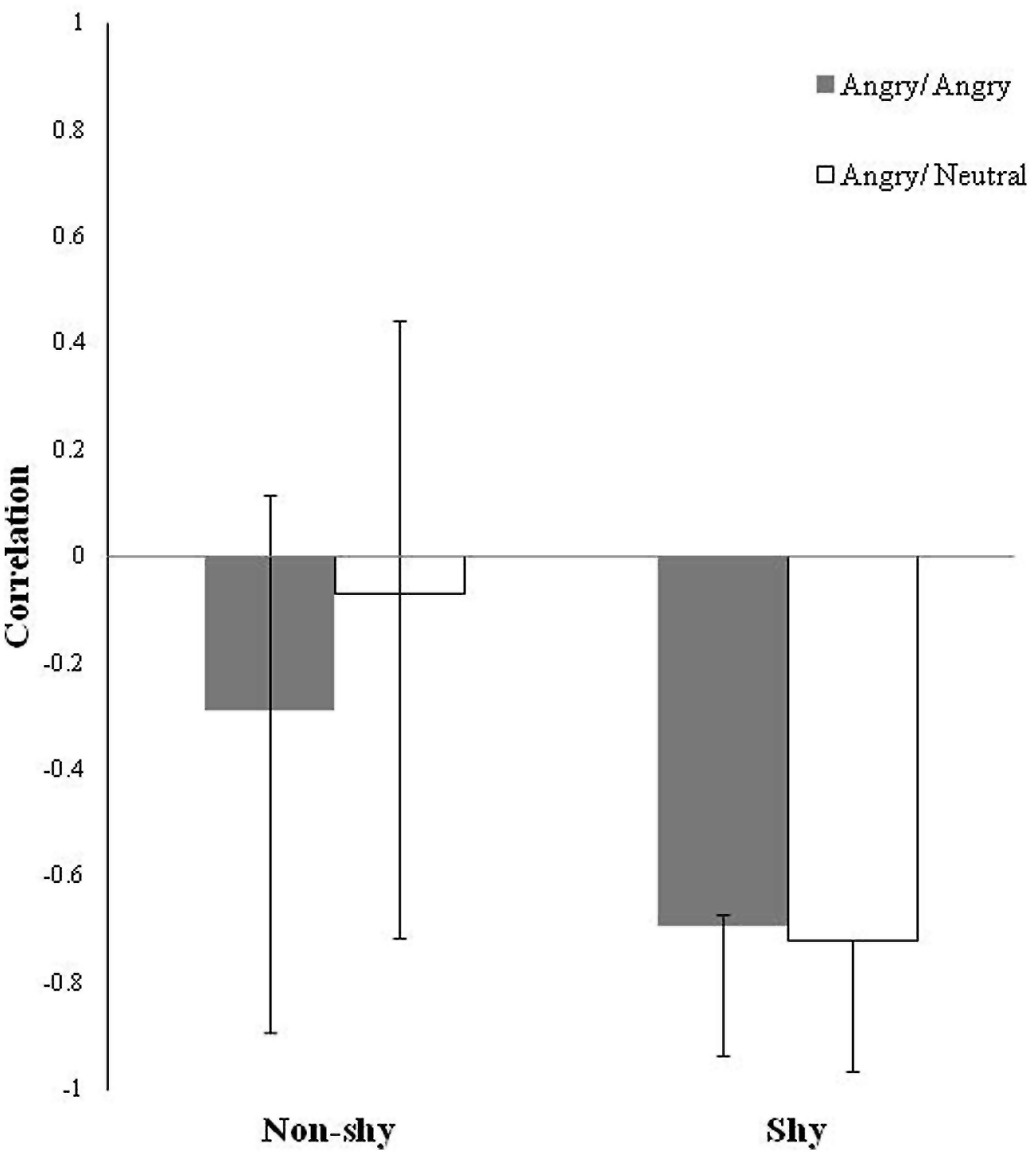
**Latent Variable (LV) pattern of brain-cortisol correlations for the two groups (non-shy, shy) and two threat conditions (imminent, ambiguous) plotted as bars.** Correlations indicate an absolute degree of relation (all positive) between brain scores (i.e., the absolute degree to which an individual expresses the identified latent brain pattern) and morning cortisol change that are task dependent. Error bars are bootstrap estimated 95% confidence intervals (CIs) for assessing the reliability of the brain-cortisol correlations. The wide CIs that crossed over zero in both threat conditions in the non-shy group indicate that those brain-cortisol correlations are not significantly different from zero, such that the identified latent brain pattern is unrelated to the cortisol pattern in the non-shy group. CIs in the shy group across both threat conditions that overlapped each other indicate the identified latent brain pattern related to the cortisol pattern in the shy group is commonly elicited to both imminent and ambiguous types of social threat.

The two group (shy, non-shy) PLS with morning salivary cortisol change score as the behavioral measure during the two types of threat conditions (imminent, ambiguous) identified one significant LV (crossblock covariance = 40.48%, *p* = 0.05). This LV revealed reliable brain-cortisol correlations for both threat conditions only in the shy group, but not for either conditions in the non-shy group as indicated by the wide confidence intervals (CIs; **Figure 1**). Here, correlations indicate an absolute degree of relation between brain score (the absolute degree to which an individual expresses the latent brain pattern) and mean morning salivary cortisol change. Reliability of correlations was assessed with regard to their corresponding CIs: non-shy, angry/angry: *r* = -0.29; CIs = -0.89 to 0.16; non-shy, angry/neutral: *r* = -0.07; CIs = -0.71 to 0.44; shy, angry/angry: *r* = -0.69; CIs = -0.94 to -0.67; shy, angry/neutral: *r* = -0.72; CIs = -0.97 to -0.72. In the non-shy group, CIs for both threat conditions included zero, indicating the correlations were not significantly different from zero, and hence did not contribute to the overall identified LV. In other words, the non-shy group did not engage in this identified network nor was this network relevant to their cortisol pattern. In the shy group, CIs for both threat conditions overlapped, indicating the brain-cortisol correlations were not significantly different between conditions. In other words, the shy group engaged in this network for imminent and ambiguous threat processing.

**FIGURE 2:**
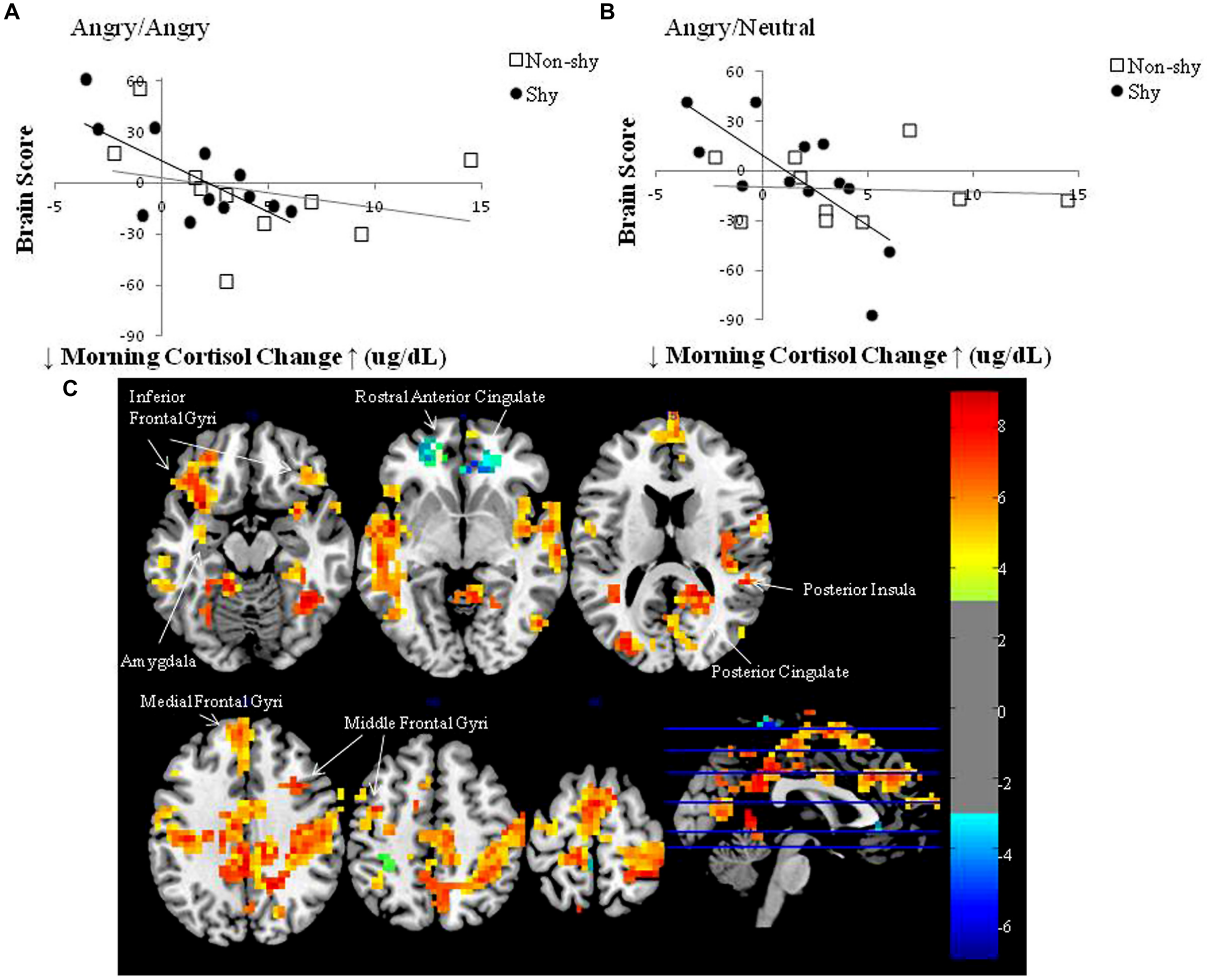
**Brain score versus mean morning cortisol change plots in the LV pattern for the two groups (non-shy, shy) and two threat conditions (imminent, ambiguous): (A) Angry/Angry; (B) Angry/Neutral; (C) Corresponding sets of brain regions**. **(A,B)** Morning cortisol change scores represent the mean CAR across three separate mornings in each individual. Brain scores represent the absolute degree to which an individual expresses the identified pattern, hence both positive and negative brain scores reflect increased activity but in different sets of regions: the top half of the graphs correspond with the set of positive saliences in **Table 1**; the bottom half of the graphs correspond with the set of negative saliences in **Table 1**. Plots **(A,B)** demonstrate that shy individuals with a lower morning cortisol change express a higher degree of relation with positive voxel saliences (corticolimbic regions in warm colors displayed in **C**), whereas shy individuals with a higher morning cortisol change express a higher degree of relation with negative voxel saliences (corticolimbic regions in cold colors displayed in **C**); also see **Table 1** for the complete set of voxel saliences) in both threat conditions. In contrast, non-shy individuals displayed weak to zero brain-cortisol correlations in Angry/Angry and Angry/Neutral conditions, respectively. **(C)** Multislice brain images displaying the two sets of corticolimbic regions that distinguished shy individuals with relatively higher and lower waking morning cortisol levels during both types of threat processing: warm colored regions increased modulation in shy adults with relatively lower morning cortisol; cold colored regions increased modulation in shy adults with relatively higher morning cortisol. Warm and cold colors are coded for the two orthogonal sets of regions shown in **Table 1**. Images were produced with bootstrap ratio (BSR) of ±3.0 (corresponding approximately to *p* < 0.003), see BSR color bar on the right side of the figure.

**Figure 2** presents the plot between brain score (the absolute degree to which an individual expresses the latent brain pattern) and mean morning salivary cortisol change for (a) imminent and (b) ambiguous threat conditions, respectively, for the two groups. The top and bottom halves of the graphs represent two orthogonal sets of regions in **Table 1**: positive saliences correspond to the upper half of the graphs; negative saliences correspond to the lower half. Relative to shy adults who have a higher CAR, shy adults who have a lower CAR also have the highest positive brain scores, which correspond to the positive saliences in **Table 1**. The set of corticolimbic regions included left amygdala, right posterior cingulate, insula, bilateral inferior, medial, and middle frontal gyri (regions in warm colors in **Figure 2**). Relative to shy adults with a lower CAR, shy adults who have a higher CAR also have the highest negative brain scores, which correspond to the negative saliences in **Table 1**. The set of corticolimbic regions included bilateral rostral ACC (regions in cold colors in **Figure 2**). In other words, while the overall LV pattern is significant for the shy group, shy adults who have the highest and lowest CAR scores exhibited the largest difference in their patterns of neural response. These results indicated relatively different functional engagement of corticolimbic regions common to both types of social threat processing among the shy group.

### RELATION BETWEEN MORNING SALIVARY CORTISOL CHANGE AND SOCIABILITY AMONG SHY ADULTS

Following the relative differential pattern of brain activity as a function of changes in waking morning salivary cortisol levels among shy adults, we assessed whether the pattern of waking morning salivary cortisol levels was related to varying levels of sociability among the shy group. The chi-square test of independence with morning salivary cortisol change score (high versus low) by sociability (high versus low) within the shy group was significant, *χ*^2^(1) = 12, *p* = 0.001. Of the 12 shy participants, 100% (i.e., 6/6) who displayed relatively higher morning salivary cortisol levels were also classified as sociable, while 100% (i.e., 6/6) who displayed relatively lower morning salivary cortisol levels were classified as unsociable, suggesting perfect classification. That is, no shy individuals who exhibited higher morning salivary cortisol levels were classified as unsociable, and no shy participants who showed lower morning salivary cortisol levels were classified as sociable.

Descriptive statistics and profiles of the CAR and sociability in the non-shy and shy groups, as well as different subtypes of shyness are shown in **Table 2**. Sociability in the shy group was significantly lower relative to the non-shy group (A). This was expected given that the two extreme groups were selected based on their shyness scores, and the shyness and sociability constructs are moderately negatively correlated (Cheek and Buss, 1981). However, shy adults with a high CAR and non-shy adults did not significantly differ on sociability; their scores were comparable (B). Accordingly, it was not the case that shy adults with a relatively higher CAR scored above average on sociability. However, shy adults with a low CAR were significantly less sociable in comparison with shy adults with a high CAR (C).

**Table 2:** Descriptive statistics and profiles of the cortisol awakening response (CAR) and sociability.

	*M* (*SD*)	
Measure	Shy	Non-shy	*t*
**(A)**
CAR	1.6 (3.07)	4.14 (4.99)	1.47
Sociability	13.5 (5.14)	17.7 (2.67)	2.33*
*p< 0.05, *df* = 20

**Measure**	**Shy with high CAR**	**Non-shy**	***t***

**(B)**
Sociability	17.33 (1.37)	17.7 (2.67)	0.31
*df* = 14			

**Measure**	**Shy with high CAR**	**Shy with low CAR**	***t***

**(C)**
Sociability	17.33 (1.37)	9.67 (4.59)	–3.92**
**p< 0.005, *df* = 10

*Independent samples *t*-tests, two tailed.*

## DISCUSSION

The present findings provide preliminary evidence of possibly relatively different neuroendocrine mechanisms underlying and maintaining shy-sociable and shy-unsociable temperamental styles. PLS demonstrated that among shy individuals, variation in morning cortisol levels were predictive of different patterns of brain activation during social threat processing, in contrast to an absence of reliable brain-cortisol correlations in non-shy individuals. This initial finding allowed further testing of the hypothesis that cortisol is related to energy expenditure that facilitates social approach- and withdrawal-related behavior (e.g., Muller and Wrangham, 2004). As predicted, shy individuals who exhibited a relatively higher CAR displayed activation in putative brain regions with modulatory roles in conflict monitoring and approach-withdrawal conflicts (e.g., ACC) during threat processing and were more sociable. In contrast, shy individuals who exhibited a relatively lower CAR displayed activation in putative brain regions involved in fear and withdrawal (e.g., the amygdala) and were unsociable.

### WHAT ARE THE LINKS AMONG HORMONE, BRAIN, AND SOCIAL BEHAVIOR?

We found that shy individuals who displayed a relatively higher CAR and bilateral rostral ACC activation scored higher on sociability. In this shy-sociable subtype, higher CAR is consistent with their social approach tendency as more energy is needed to be socially outgoing. As well, although the dorsal ACC has been more reliably observed in conflict monitoring, both dorsal and rostral aspects are modulated in conflict monitoring of emotional stimuli (for a review, see Etkin et al., 2011). Furthermore, increased modulation of the rostral ACC is related to individual differences in more intense experience of emotion or emotion awareness (Lane et al., 1998). As shy-sociable individuals are motivated to keep track of others’ emotions while dealing with their own emotional experience during social challenges, these functions of the ACC are consistent with their known approach-avoidance conflict (Coplan et al., 2004), and right frontal EEG asymmetry and hyperactivity in both the left and right PFC that is believed to result in the experience of intense negative emotions (Schmidt, 1999).

Shy adults who displayed a relatively lower CAR and higher activation of left amygdala, right posterior cingulate, insula, bilateral inferior, medial, and middle frontal gyri – scored lower on sociability. In this shy-unsociable subtype, lower CAR may facilitate social withdrawal and reflect an adaptation of the neuroendocrine system to dealing with life-long stress of being shy. The stronger relation with left amygdala activation, a region associated with fear-related emotions (Phelps and LeDoux, 2005) may be linked to fear of negative social evaluation and threat detection (Larson et al., 2009), which fit with their withdrawal-related behavior. Also, regions that are abnormal in mood and anxiety disorders, including the amygdala, posterior cingulate, and insula (for a review see, Price and Drevets, 2012) were more related to shy-unsociable individuals, which corroborates the risk of developing social phobia and depression in this subtype.

Notably, the attenuated CAR observed in shy-unsociable adults is similar to “burnout” observed in other stressed profiles (Gunnar and Vazquez, 2001). Such low levels have been associated with cognitive vulnerability to depression and social withdrawal in non-clinical young adults (Kuehner et al., 2007), and with overactivity or underactivity of the HPA-axis depending on the severity and subtype of depression (Oswald et al., 2006). It is conjectured that the combination of reduced morning waking cortisol levels and increased amygdala activation to social threat in shy-unsociable adults potentially contribute to increased corticotropin-releasing hormone levels and adaptation of the HPA-axis to dealing with social anxiety across development.

### LIMITATIONS AND CLARIFICATION

There are at least three limitations that warrant discussion. First, although an extreme group design was used, the present preliminary results were based on a relatively small sample and statistical analyses that might have capitalized on the small sample. Second, the number of trials for the two threat-related conditions was also relatively small and no positive stimuli were incorporated. Third, although the CAR has been demonstrated to peak within the first 30 min upon awakening and remain relatively stable up to 60 min (Wust et al., 2000), our two morning cortisol samples each collected across 3 days may not have completely characterized the morning CAR response. Accordingly, future studies need to replicate the present preliminary results using a larger sample size, and more trials using threatening and positive stimuli, and repeated measures of morning salivary cortisol within the first hour of waking in order to ensure the reliability and generalizability of the present preliminary findings.

Finally, it is also important to point out that the current focus was to delineate brain signals to different kinds of social threat as they relate to individual differences in shyness and cortisol patterns. Due to the data-driven nature of PLS, although data for both shy and non-shy participants were inputted into PLS, the only significant and reliable latent brain pattern to both types of social threat (imminent and ambiguous) that related with cortisol patterns was identified in the shy group (as discussed in **Figure 1**). This means there was no systematic latent brain pattern to social threat processing in the non-shy group that was relevant to non-shy individuals’ cortisol patterns. Furthermore, because the latent brain pattern in the shy group was not different across the two social threat conditions, it follows that the discrimination nature of the task could not have driven these results (for different task activation to the two types of threat common in both shy and non-shy groups that was unrelated with cortisol; see Tang et al., under review), rather, aspects of social threat and/or negative emotion inherent in both imminent and ambiguous social threat stimuli might have contributed to the results.

## CONCLUSION

The current preliminary findings suggest that not all people who are shy are alike. We speculate that individual differences in morning cortisol levels among shy individuals may bias perceptual and/or psychological frameworks in the brain that facilitate social approach and social withdrawal to thereby explain heterogeneity in the shyness phenomenon. Considering these results within a diathesis-stress framework, different brain-cortisol patterns may reflect separate diatheses for the shy-sociable and shy-unsociable subtypes that are manifested differently during social challenges – the former resulting in an approach-withdrawal conflict, and the latter resulting in social withdrawal. We also speculate that these putative brain-endocrine diatheses may underlie the cascade of secondary negative effects of social anxiety/substance use and depression observed in shy-sociable and shy-unsociable subtypes, respectively. Nevertheless, causal inferences cannot be drawn from the current non-directional associations among these hormone-brain-behavior relations. Future longitudinal studies should model variables relating to environmental and age-related factors and dynamics among different biological and behavioral variables to explain how different types of shyness develop or change over time in order to improve on causal inferences with a larger sample.

## Conflict of Interest Statement

The authors declare that the research was conducted in the absence of any commercial or financial relationships that could be construed as a potential conflict of interest.
